# CNI Trough Variability Does Not Reliably Reflect Medication Adherence: Insights From a 3-Year Follow-Up Study

**DOI:** 10.3389/ti.2026.15718

**Published:** 2026-03-02

**Authors:** Claire Villeneuve, Jean-phillipe Rerolle, Lionel Couzi, Pierre-Francois Westeel, Isabelle Etienne, Laure Esposito, Nassim Kamar, Mathias Büchler, Antoine Thierry, Pierre Marquet, Caroline Monchaud

**Affiliations:** 1 CHU Limoges, Department of Pharmacology, Toxicology and Centre of Pharmacovigilance, Limoges, France; 2 INSERM, UMR-1248, Limoges, France; 3 FHU SUPORT, Limoges, France; 4 Department of Nephrology, Dialysis and Transplantation, Limoges, France; 5 Department of Nephrology, Transplantation, Dialysis, Centre Hospitalier Universitaire (CHU) Pellegrin, Bordeaux, France; 6 CNRS-UMR 5164 Immuno ConcEpT, Bordeaux University, Bordeaux, France; 7 Department of Nephrology and Kidney Transplantation, University Hospital of Amiens, Amiens, France; 8 Service de Nephrologie, Rouen University Hospital, Rouen, France; 9 Department of Nephrology and Organ Transplantation, CHU Toulouse, Toulouse, France; 10 INFINITY-Inserm U1291-CNRS U5051 University Paul Sabatier, Toulouse, France; 11 INSERM U1043, IFR-BMT, CHU Purpan, Toulouse, France; 12 Department of Nephrology and Transplantation, University of Tours, Tours, France; 13 Équipe d'accueil4245, François Rabelais University, Tours, France; 14 CHU Poitiers, Department of Nephrology, Dialysis and Transplantation, Poitiers, France; 15 University Limoges, Faculty of Medicine, Limoges, France

**Keywords:** adherence, calcineurin inhibitor (CNI), kidney transplantation, trough concentrations (C_0_), variability

## Abstract

Calcineurin inhibitor (CNI) trough concentrations and their variability are frequently used as adherence proxies, despite limited validation. We evaluated the association between self-reported adherence and CNI exposure during the first year after kidney transplantation. We included 619 patients from two prospective French cohorts (14,829 C_0_ values). Adherence was assessed using the MMAS-4 questionnaire. CNI exposure was evaluated via C_0_ levels, intra-patient variability (IPV; CV threshold = 30%), and underexposure rates. Cross-sectional and longitudinal analyses were performed. No significant differences in C_0_, IPV, or underexposure were observed between adherent and non-adherent patients, regardless of the CNI used or analytical approach. In longitudinal analysis, IPV was similar (31.3% [25.5–38.2] vs. 31.6% [23.6–38.9], p = 0.68), as was the proportion of patients with high IPV (55.5% vs. 51.5%, p = 0.5). At 3 years, high IPV was not significantly associated with rejection (HR 1.02 [0.67–1.55], p = 0.93). Self-reported adherence was not associated with CNI C_0_ levels, IPV, or underexposure. CNI C_0_ variability alone cannot reliably detect non-adherence and should not be interpreted as a standalone adherence marker. Multimodal strategies combining pharmacokinetics with validated self-report tools are needed to evaluate adherence.

## Graphical Abstract

**Figure:**
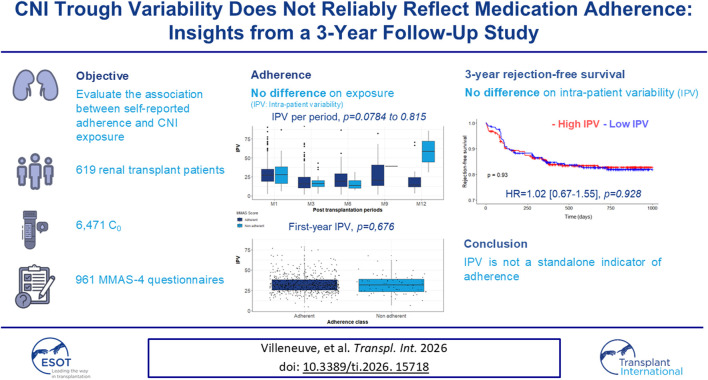

## Introduction

Over the past years, medication adherence has emerged as a major concern for clinicians, policymakers and health managers. In kidney transplantation, the prevalence of poor adherence ranges between 15% and 43% in adults and between 40% and 60% in adolescents [1–7]. It is a critical determinant of post-transplant outcomes, contributing to late rejection, chronic allograft dysfunction, nephropathy and ultimately, graft loss [1, 8, 9]. It is also associated with psychosocial complications, reduced quality of life, and substantial healthcare costs. Consequently, improving adherence may yield benefits comparable to those of optimized or new pharmacological interventions [10].

Conducting studies on adherence requires clear definitions and robust and validated measurement tools. The World Health Organization originally defined adherence as “the extent to which a person’s behavior–taking medication, following a diet, and/or executing lifestyle changes, corresponds with agreed recommendations from a healthcare provider” [10]. The concept of medication adherence has since evolved to encompass a dynamic process, comprising initiation, implementation and discontinuation [11]. Nevertheless, no gold standard for adherence measurement has been established to date [12]. A wide variety of tools have been employed in transplant populations, including pill counts, pharmacy refill databases, Medication Electronic Monitoring Systems (MEMS), immunosuppressant blood concentrations and validated questionnaires such as the BAASIS, ITAS or MMAS [9, 13–15]. Composite scores combining questionnaires and pill count or drug concentrations have also been used [14, 16].

Among these tools, trough concentrations (C_0_) of calcineurin inhibitors (CNIs), particularly tacrolimus, have been proposed as a proxy of adherence. Several approaches have been proposed: (i) evaluating intra-patient variability (IPV) via coefficient of variation over a period [9, 13, 14, 17, 18]; (ii) assessing mean C_0_ levels [19]; (iii) estimating the proportion of C_0_ values outside of the therapeutic range [13, 20]; (iv) calculating the time spent outside of the therapeutic range [14, 21]. While exposure to immunosuppressants and its variability are known to influence transplant patients outcomes [18, 22–24], the association between drug exposure biomarkers and non-adherence remains inconsistent. Most studies rely on cross-sectional designs whereas non-adherence is a dynamic phenomenon that may be isolated or repeated over time [1]. In this regard, longitudinal analysis of drug exposure variability may better reflect therapeutic implementation patterns. However, correlating long-term variability with a single-point questionnaire is methodologically questionable. Moreover, the pharmacokinetics of tacrolimus is influenced by numerous factors, and the proportion of variability attributable solely to non-adherence is likely to be small.

In clinical practice, poor adherence can be suspected when tacrolimus concentrations are unexpectedly low. However, in research settings, the validity of using drug levels as surrogate adherence markers remains questionable. Despite this, both clinicians and researchers continue to infer adherence behaviors from drug exposure data [12, 18], and C_0_ monitoring remains a widely used -albeit imperfect- proxy [25].

In this context, the objective of our study was to explore the relationship between adherence and exposure to calcineurin inhibitors (CNIs) during the first year after kidney transplantation. Because the clinical consequences of high CNI intra-patient variability–whether driven by non-adherence or other factors–may only emerge later, we also examined the association between first-year CNI exposure and graft rejection occurring up to 3 years post-transplantation.

## Materials and Methods

### Design and Study Population

This study included patients from two successive multicenter prospective cohorts: EPIGREN, conducted between 2007 and 2011 in three French kidney transplantation centers, and EPHEGREN, conducted between 2012 and 2017 in the same centers plus four additional ones. Both studies were sponsored by Limoges University Hospital (CHU Limoges) and complied with the legal requirements of the Declaration of Helsinki. Regulatory approvals were received from the French Medicine Agency (authorization no. 060566, dated 08/08/2006), the French National Data Protection Authority (CNIL; no. 907275 ACT in 2006 for EPIGREN, no. 912242 ACT in 2012 for EPHEGREN) and the relevant regional Ethics Committee (no.06-040 on 19/05/2006 for EPIGREN and no. 130-2013-30 on 20/11/2013 for EPHEGREN).

All *de novo* kidney transplant patients followed-up in the participating centers were eligible for inclusion, except if they met the following non-inclusion criteria: (i) inability to understand written French; (ii)inability to understand the protocol; (iii)impossibility to be followed in one of the seven investigating centers (refusal of regular long-term follow-up, moving, etc.). Eligible patients were enrolled within the first month post-transplantation during a routine consultation, after receiving information about the study and giving their written consent.

**FIGURE 1:**
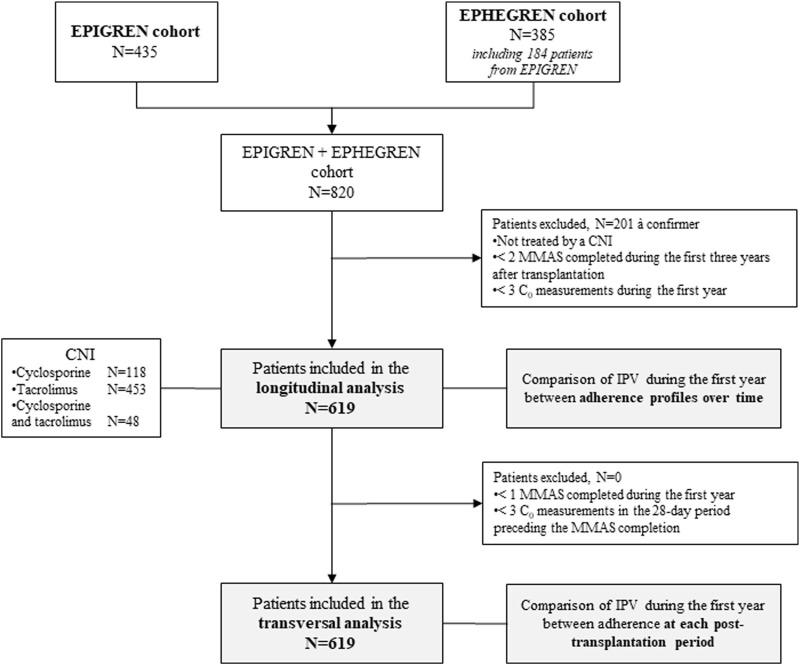
Flowchart of the study.

A total of 820 patients were recruited in these two cohorts (Figure 1). Their immunosuppressive regimens were left to the discretion of the investigators and complied with the standard of care for transplanted patients (mainly CNIs -either tacrolimus or cyclosporine- in combination with mycophenolate). Dose adjustments of CNIs were based on trough concentrations, using the recommended target ranges [22, 26, 27].

#### Data Collection

Following inclusion, patients were scheduled for up to eight visits over a three-year period after the transplantation: at 1 month (M1), 3 months (M3), 6 months (M6), 12 months (M12), 24 months (M24), and 36 months (M36). An additional visit at 9 months (M9) was included for patients in the EPIGREN cohort.

At each visit, clinical outcomes -including graft rejection- and biological data, were collected from the medical records and patient-reported outcomes assessed using self-administered questionnaires.

### Self-Reported Adherence

Self-reported adherence was assessed using the 4-Item Morisky-Green-Levine Medication Adherence Scale (MMAS-4) (Supplementary Material) [28]. The MMAS-4 is straightforward to administer and interpret, has been formally validated in French and among transplant populations, and is well suited for repeated longitudinal assessments [1]. The questionnaire was self-administered during scheduled follow-up visits in accordance with the study protocol and completed by patients without assistance from clinicians or family members. Participants filled out the questionnaire in the waiting room prior to their visit. These research visits were synchronized with routine clinical follow-up. A score of 0 was considered indicative of adherence, whereas any score greater than 0 indicated non-adherence.

Both cross-sectional and longitudinal approaches were employed in this study. Therefore, only patients who had completed the MMAS-4 at least twice during the three-year post-transplantation follow-up period were considered (Figure 1). In the cross-sectional approach, adherence was evaluated independently at each follow-up visit. In the longitudinal approach, patients were categorized into two groups based on their previously established adherence trajectories over time, identified using a mixed-effects modeling framework with latent processes and latent classes, as described by Villeneuve et al. [1] (Supplementary Material). The two resulting categories were: (i) patients with a consistently good and stable adherence over time; (ii) patients with a poor and worsening adherence.

### Exposure to Calcineurin Inhibitors

Both IPV and underexposure to CNIs were analyzed. To be eligible for this analysis, patients had to have at least three C_0_ measurements of either cyclosporine or tacrolimus over the first year post-transplantation. Patients not meeting this criterion were excluded (Figure 1). In addition, extreme C_0_ values (≥500 μg/L for cyclosporine and ≥20 μg/L for tacrolimus) were considered outliers and excluded from the dataset.

IPV was quantified using the coefficient of variation (CV), calculated as the ratio of the standard deviation (SD) to the mean (μ) of C_0_ values. Variability was classified as high (CV ≥30%) or low (CV <30%), in line with previously published thresholds [17]. Underexposure was assessed based on the proportion of C_0_ values below the lower bound of the therapeutic window. For cyclosporine, these thresholds were 200 μg/L at M1, 150 μg/L between M2 and M6 and 100 μg/L beyond M6. For tacrolimus, a fixed threshold of 4 μg/L was applied across all time points [22, 26, 29].

### Missing Data Management

CNI trough levels were obtained as part of routine clinical care and were considered exhaustive for the study period, as all trough concentrations available from routine therapeutic drug monitoring during follow-up were included in the analyses. For the MMAS, patients were invited to complete the questionnaire at each visit. For the longitudinal analysis, we used mixed models with latent processes and latent classes to characterize adherence profiles over time. This modeling framework accommodates incomplete longitudinal observations. Parameters were estimated by maximum likelihood, under the assumption that missing data were missing at random.

### Statistical Analysis

All analyses were performed using R version 4.2(R Foundation for Statistical Computing: http://www.r-project.org). Categorical variables are reported as frequencies and percentages, and continuous data as their median and interquartile range [IQR] otherwise. Group comparisons were performed using Pearson’s chi-square or Fisher’s exact tests for categorical data, and Student’s t-test or Kruskal-Wallis test for continuous variables, as appropriate. Bonferroni risk correction was applied in the case of multiple comparisons.

The relationship between adherence and CNI exposure was explored using both cross-sectional and the longitudinal approaches. In the cross-sectional analysis, adherence at each visit (based on MMAS-4) was compared to C_0_ values collected during the preceding 28-days. In the longitudinal analysis, adherence profiles were compared to C_0_ values collected during the first year after transplantation. Both analyses were performed for the entire cohort and for each CNI. To ensure the robustness of intra-patient variability (IPV) estimation, a minimum of three trough concentrations per analysis window was required. A single IPV estimate was calculated for each patient and each analysis window, ensuring that each individual contributed only one IPV value, irrespective of the number of available measurements.

The relationship between IPV and graft rejection at 3 years post-transplantation was explored using a Cox proportional hazards model. An initial univariate analysis was conducted, followed by inclusion of variables with *p* < 0.20 in a multivariable model. Final model selection was performed using backward stepwise elimination based on the Bayesian Information Criterion (BIC). Model robustness was assessed through 1,000 bootstrap resamples, each followed by backward stepwise selection using the same procedure. The hazard ratios (HR) and 95% confidence intervals (95% CI) derived from the final model and the percentage of selection of each covariate in the bootstrap procedure were calculated [30] Candidate covariates included recipient-related, treatment-related, and immunological variables routinely collected in the cohort. Time-to-rejection was estimated using Kaplan-Meier survival analysis and high IPV vs. low IPV patients were compared using the log-rank test. The proportionality of risks in the final models was verified using the Schoenfeld residuals.

Two-sided p-values <0.05 were considered to be statistically significant.

## Results

### Description of the Population

A total of 619 patients were included in this study, contributing to 14,829 C_0_ measurements during the first year post-transplantation (Figure 1). The main sociodemographic and clinical characteristics of the cohort are summarized in Table 1.

**TABLE 1:** Characteristics of the cohort (N = 619). Continuous variables are reported in median [IQR], categorial variables are reported in numbers (%).

N	619	Missing data (%)
Age (years)	54.0 [44.5–64.0]	0.0
Gender, male	398 (64.3)	0.0
Height (cm)	170 [163–176]	29.4
Weight (kg)	72 [62–82]	1.3
Body Mass index	25.1 [22.0–28.3]	29.4
Occupational status	​	20.7
Active	86 (17.5)	​
Retired or without any professional activity	387 (78.8)	​
Other	18 (3.7)	​
Rank of kidney transplantation	​	0.0
0	535 (86.4)	​
1	78 (12.6)	​
2	6 (1.0)	​
Primary kidney disease	​	25.2
Glomerulonephritits	138 (29.8)	​
Genetic disease	129 (27.9)	​
Vascular nephropathy	43 (9.3)	​
Interstitial nephritis	38 (8.2)	​
Diabetic nephropathy	22 (4.8)	​
Others	93 (20.1)	​
History of hypertension before transplantation	455 (92.3)	20.4
History of diabetes before transplantation	64 (13.0)	20.4
Immunosuppressive strategy	​	0.0
Cyclosporine	118 (19.1)	​
Tacrolimus	453 (73.2)	​
Switch cyclosporine/tacrolimus	48 (7.8)	​
Patients on MMF (%)	603 (97.4)	0.0
MMF formulation (%)	​	2.6
Cellcept	411 (68.2)	​
Myfortic	144 (23.9)	​
Cellcept/Myfortic	48 (8.0)	​
Patient therapeutic education, before transplantation	175 (35.5)	20.4
Patient therapeutic education, after transplantation	120 (24.3)	20.4
Functional graft at the end of study	476 (96.6)	20.4

Among the participants, 118 patients received cyclosporine only, 453 tacrolimus only, and 48 received both CNIs at different times during the first year (Supplementary Table S1; Supplementary Figure S1). Almost all patients were also treated with mycophenolate as part of their immunosuppressive regimen (Supplementary Table S2).

The median [IQR] C_0_ was 8.6 [6.8–10.6] μg/L for tacrolimus (11,618 C_0_ values) and 161 [124–217.0] μg/L for cyclosporine (3,211 C_0_ values). Outlier values, as defined in the Methods, represented less than 1% of cyclosporine concentrations and less than 2% of tacrolimus concentrations.

### Cross-Sectional Approach

#### Description of Adherence and Exposure Data

At least three C_0_ measurements were available during at least one 28-day period preceding MMAS-4 administration in 619 patients, who were included in this analysis (Figure 1). This dataset comprised 961 adherence questionnaires and a total of 6,471 C_0_ values – 4,252 for tacrolimus and 1,225 for cyclosporine (Table 2). The median [IQR] number of C_0_ values per 28-day period decreased over time, from 7 [5–8] in the first month to 3.5 [3, 4] between M6 and M12.

**TABLE 2:** Comparison of IPV (whatever the CNI) in adherent vs. non-adherent patients according to follow-up periods after the transplantation, using the cross-sectional approach. Continuous variables are presented in median [IQR] and categorial variables are presented in numbers. Continuous variables are compared using Wilcoxon test and categorial variables are compared using Chi^2^ test.

Period	Status	Patients (n)	IPV median [IQR]	p	High IPV(n)	p	% C_0_	p
a. Overall								
M1	A	490	27.2 [19.2–35.0]	0.82	190	0.86	33.3 [19.2–57.1]	**0.04**
	NA	21	27.6 [16.2–38.4]		12		17.1 [11.5–36.5]	
M3	A	306	15.7 [10.3–24.6]	0.61	43	0.67	50.0 [25.0–100.0]	0.65
	NA	27	15.8 [12.1–20.1]		3		75.0 [40.0–80.0]	
M6	A	80	19.2 [12.2–29.1]	0.17	19	0.63	87.5 [50.0–100.0]	0.40
	NA	7	13.6 [9.8–20.2]		1		83.3 [75.0–91.7]	
M9	A	11	20.6 [13.5–40.7]	0.67	4	0.86	50.0 [43.8–62.5]	0.81
	NA	1	39.1 [39.1–39.1]		1		45.0 [42.5–47.5]	
M12	A	16	14.1 [11.6–24.3]	0.08	3	0.11	100 [68.8–100]	0.34
	NA	2	58.3 [44.5–72.0]		2		50.0 [50.0–50.0]	

Period	Group	Patients (n)	Number of C_0_	C_0_ median [IQR] (µg/L)	p	IPV median [IQR] (%)	p	High IPV (n)	p	% C_0_ < target median [IQR]	p
b. For cyclosporin											
M1	A	113	820	214 [157–273]	0.71	27.6 [21.1–40.4]	0.46	49	0.83	55.6 [33.3–77.8]	0.36
	NA	4	32	221 [178–268]		22.1 [13.9–34.9]		1		37.5 [20.0–60.0]	​
M3	A	63	263	166 [128–211]	0.16	15.9 [10.9–27.5]	0.53	11	1.00	75.0 [33.3–100.0]	0.44
	NA	6	24	144 [121–180]		14.0 [12.8–16.0]		1		80.0 [75.0–100.0]	​
M6	A	15	56	145 [122–198]	0.84	26.3 [10.8–35.0]	0.63	6	1.00	100.0 [50.0–100.0]	0.82
	NA	1	3	148 [138–154]		11.1 [11.1–11.1]		0		66.7 [66.7–66.7]	​
M9	A	2	6	145.0 [138.0–187.2]	0.17	52.1 [37.2–67.0]	1.00	1	1.00	50.0 [50.0–50.0]	NA
	NA	1	4	101.5 [83.8–122.5]		39.1 [39.1–39.1]		1		50.0 [50.0–50.0]	​
M12	A	4	17	122.0 [97.0–147.0]	Not estimable	23.0 [15.5–37.5]	Not estimable	1	Not estimable	100.0 [93.8–100.0]	Not estimable
	NA	0	0	–		–		0		–	​
c. For tacrolimus											
M1	A	370	2576	8.9 [6.9–11.1]	0.04	26.9 [18.3–34.4]	0.87	141	0.59	20.0 [12.5–33.3]	0.74
	NA	24	171	9.6 [7.1–11.8]		27.8 [18.1–38.4]		11		14.3 [12.5–33.3]	
M3	A	239	1031	8.8 [7.4–10.5]	0.93	15.7 [10.2–24.1]	0.87	32	0.65	29.2 [20.0–52.5]	Not estimable
	NA	25	111	8.8 [7.6–10.3]		16.4 [11.7–21.0]		2		0	
M6	A	63	243	8.2 [6.1–10.1]	0.99	17.1 [12.3–28.4]	0.31	13	0.94	66.7 [50.0–100.0]	0.36
	NA	8	31	8.1 [6.7–9.2]		14.3 [9.3–22.5]		1		100.0 [100.0–100.0]	
M9	A	9	35	8.0 [6.1–10.4]	Not estimable	19.1 [12.4–37.3]	Not estimable	3	1.00	62.5 [43.8–81.3]	Not estimable
	NA	0	0	–		–		0		–	
M12	A	12	47	6.9 [5.5–9.2]	0.58	13.8 [11.6–18.7]	0.09	2	0.12	100.0 [38.3–100.0]	0.832
	NA	2	7	7.2 [4.2–8.4]		58.3 [44.5–72.0]		2		50.0 [50.0–50.0]	

Bold values indicate statistical significance (p < 0.05).

The proportion of patients classified as non-adherent tended to increase over time, rising from 4.1% during the first month to 11.1% between M9 and M12 (Table 2). However, this trend did not reach statistical significance across the 5 follow-up periods (p = 0.83).

No significant difference in median C_0_ values was observed between adherent and non-adherent patients, regardless of the period or the CNI administered (Table 2).

#### Relationship Between Adherence and Intra-patient Variability

The median IPV, as well as the proportion of patients with high IPV (CV ≥30%) were not significantly different between post-transplant periods (Table 2). Furthermore, no significant differences in IPV (median CV or proportion with high IPV) were found between adherent and non-adherent patients during any of the evaluated time periods (Table 2).

#### Relationship Between Adherence and Underexposure

The proportion of C_0_ values below the therapeutic target in the 28 days preceding each MMAS-4 assessment did not differ significantly between adherent and non-adherent patients at any post-transplantation time point. At M1, the proportion of subtherapeutic C_0_ values for each patient was higher in adherent patients compared to non-adherent patients (33.3% [IQR: 19.2–57.1] vs. 17.1% [11.5-36.5] (p *=* 0.04). However, this difference was not significant when considering patients on cyclosporine and tacrolimus separately. Moreover, no significant differences were found either between adherent and non-adherent patients from M3 to M12 (Table 2). Overall, these findings suggest that underexposure to CNIs was comparable in both groups and was not significantly associated with self-reported adherence at any time point.

### Longitudinal Approach

#### Description of Adherence and Exposure Data

Based on longitudinal adherence profiling, the majority of patients (551/619) were classified as adherent over time.

There were no significant differences in baseline characteristics between adherent and non-adherent patients, with the exception of age (p < 0.001) and pre-transplant diabetes (p = 0.024): non-adherent patients were significantly younger than adherent patients and had more frequently pre- transplant diabetes.

When stratified by CNI, the patients classified in the non-adherence class where respectively 42 on tacrolimus, 10 on cyclosporine and 6 who switched from one CNI to the other.

No significant differences in median C_0_ values over the first year were observed between adherent and non-adherent patients for either tacrolimus (8.6 [6.8–10.6] vs. 8.7 [6.9–10.6] µg/L, p = 0.434) or cyclosporine (163 [124–218] vs. 155 [122–202] µg/L, p = 0.117). This absence of difference was consistent across most follow-up periods, except at M6 and M12 for patients on tacrolimus.

#### Relationship Between Adherence and Intra-patient Variability

The overall IPV did not differ significantly between adherent and non-adherent patients: 31.3% [25.5–38.2] vs. 31.6% [23.6–38.9], p = 0.676. Similar findings were observed when stratified by CNI: for cyclosporine, the median IPV was 32.8% [26.8–42.9] in adherent vs. 36.9% [26.1–45.5] in non-adherent patients (p = 0.531), and for tacrolimus, the median IPV was 31.0% [25.0–37.4] in adherent vs. 30.7% [22.5–37.4] in non-adherent patients (p = 0.459).

The proportion of patients with high IPV was also comparable between groups: 55.5% in adherent vs. 51.5% in non-adherent patients (p = 0.5). Similar results were found when examined by CNI: 59.3% in adherent vs. 68.8% in non-adherent patients on cyclosporine (p = 0.5) and 54.0% in adherent vs. 50.0% in non-adherent patients on tacrolimus (p = 0.6).

#### Relationship Between Adherence and Underexposure

Overall, the proportion of C_0_ values below the lower limit of the therapeutic window was comparable between adherent and non-adherent patients (11.7% vs. 10.3%, p = 0.12). When stratified by treatment, a significant difference was observed among patients on tacrolimus (2.6% in adherent vs. 3.8% in non-adherent patients, p = 0.026), whereas no significant difference was found for cyclosporine (39.3% vs. 39.7%, p = 0.935).

### CV and Rejection

**FIGURE 2:**
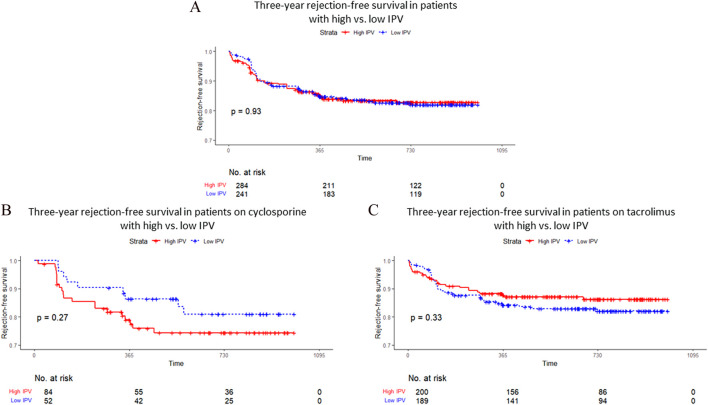
Survival without rejection over the 3 first years post-transplantation (CV >30% vs. CV <30%), globally **(A)**, for cyclosporine **(B)** and for tacrolimus **(C)**.

Rejection-free survival at 3 years post-transplantation did not differ significantly between patients with low versus high IPV during the first year, both overall and when stratified by type of CNI (HR _low_ = 1.02 [0.67–1.55], p = 0.928, Figure 2). In multivariate Cox analysis, rejection-free survival was significantly associated with HLA mismatch and *de novo* DSA (both associated with poorer outcomes) whereas therapeutic patient education was independently associated with improved outcomes (Table 3).

**TABLE 3:** Univariate and multivariate Cox analyses of the association of rejection-free survival and potential risk factors over the first 3 years post-transplantation.

Characteristic	N	HR^a^	CI 95%	p value	HR	CI 95%	p value	% Bootstrap selection
Age (years)	525	0.99	0.98, 1.01	0.361	​	​	​	​
Gender (male)	525	0.96	0.62, 1.48	0.840	​	​	​	​
Rank of kidney transplantation (vs. 0)	525	​	​	​	​	​	​	​
1	​	1.28	0.69, 2.35	0.433	​	​	​	​
2	​	1.57	0.22, 11.3	0.656	​	​	​	​
Functional graft at the end of study (YES)	395	0.08	0.04, 0.15	<0.001	​	​	​	​
MMF speciality (vs. Cellcept)	511	​	​	​	​	​	​	​
Switch Cellcept to myfortic	​	1.90	0.97, 3.74	0.062	​	​	​	​
Myfortic	​	0.98	0.60, 1.59	0.933	​	​	​	​
First-line CNI (vs. cyclosporine)	525	​	​	​	​	​	​	​
Switch (cyclosporine to tacrolimus)	​	2.39	1.28, 4.48	0.007	​	​	​	​
Tacrolimus	​	0.81	0.45, 1.46	0.492	​	​	​	​
IPV (low)	525	1.02	0.67, 1.55	0.928	​	​	​	​
Pre-transplant hypertension (yes)	395	0.57	0.21, 1.59	0.285	​	​	​	​
Pre-transplant diabetes (yes)	395	0.36	0.11, 1.15	0.083	​	​	​	​
Pre-transplant therapeutic education (yes)	**395**	**0.55**	**0.30, 0.98**	**0.043**	**0.47**	**0.24, 0.91**	**0.026**	**85.5**
Post-transplant therapeutic education (yes)	395	1.18	0.66, 2.11	0.586	​	​	​	​
Adherence class (non-adherent)	525	0.96	0.46, 1.98	0.902	​	​	​	​
HLA mismatch	**433**	**1.31**	**1.11, 1.53**	**<0.001**	**2.35**	**1.18, 4.65**	**0.014**	**76.0**
Cold ischemia time >1000 (yes)	437	1.00	1.00, 1.00	0.201	​	​	​	​
Delayed graft function (yes)	525	0.74	0.36, 1.53	0.420	​	​	​	​
Pre-transplant DSA (yes)	525	1.37	0.63, 2.96	0.428	​	​	​	​
*De novo* DSA (yes)	**456**	**2.40**	**1.42, 4.06**	**0.001**	**1.23**	**1.01,1.50**	**0.044**	**79.0**

^a^ HR, Hazard Ratio; CI, Confidence Interval. Bold values indicate statistical significance (p < 0.05).

## Discussion

Several previous studies have suggested an association between immunosuppressant C_0_ and medication adherence. In the present study, we tested this hypothesis using data from 619 kidney transplant recipients enrolled in two prospective cohorts, and who benefited from regular follow-up visits from M1 to M36 post-transplantation. Our analysis focused primarily on CNI exposure data collected during the first year, a period during which adherence was also repeatedly assessed. Both cross-sectional and longitudinal approaches were applied, allowing for a detailed evaluation of these two dynamic and time-dependent phenomena. Adherence was measured using the MMAS-4 questionnaire, while exposure to CNIs was assessed through C_0_, intra-patient variability (IPV), and the proportion of subtherapeutic C_0_ values.

In the cross-sectional approach, C_0_ were compared between adherent (MMAS = 0) and non-adherent (MMAS >0) patients at each follow-up visit during the first year. In the longitudinal analysis, adherence profiles were derived from MMAS-4 data collected over the full three-year follow-up period, while CNI exposure was restricted to the first year post-transplantation. Across both analytical approaches, no significant differences were observed in C_0_ values, IPV, or the proportion of subtherapeutic C_0_ measurements between adherent and non-adherent patients.

Our findings are consistent with several previous studies that questioned the reliability of CNI trough concentrations as a proxy for adherence. For example, Reese et al. used electronic monitoring of pill bottle openings to assess adherence in a study comparing a control group to two intervention groups receiving reminder notifications. While non-adherence rates differed significantly between groups, no differences were observed in mean tacrolimus concentrations [31]. Similarly, Schäfer-Keller et al. used electronic monitoring as the reference standard and evaluated various methods for detecting non-adherence, including immunosuppressant blood level monitoring. None of the tested methods, however, demonstrated acceptable sensitivity or specificity [32].

Leino et al. applied a previously proposed cutoff for tacrolimus IPV to identify patients at high risk of non-adherence but found no significant differences in adherence between patients with high versus low IPV [33]. This was likely due to a highly adherent study population with limited variability in tacrolimus exposure. In our study, we similarly observed no significant difference in the proportion of patients with high CNI IPV between adherent and non-adherent groups.

Scheel et al., using the BAASIS questionnaire -which is conceptually comparable to the MMAS-4 and ITAS- found that rejection was associated with patient-reported non-adherence and the percentage of subtherapeutic C_0_. However, they did not observe any association between non-adherence and either IS concentration variability or the percentage of subtherapeutic levels [13]. Similarly, several other studies using questionnaires (BAASIS or ITAS), often combined with interviews or electronic monitoring (MEMS), have reported no significant differences in CNI IPV between adherent and non-adherent patients [9, 13, 14, 16]. Our results support these findings, as neither IPV nor the proportion of subtherapeutic C_0_ values differed significantly between patients classified as adherent and non-adherent. This conclusion holds true for patients treated with tacrolimus. However, in the subgroup receiving cyclosporine, adherent patients exhibited higher IPVs, possibly reflecting the well-documented higher pharmacokinetic variability associated with cyclosporine.

McGillicuddy et al. explored whether a mobile health intervention could improve adherence and observed a reduction in tacrolimus IPV along with improved clinical outcomes [25]. However, it remains unclear whether these benefits were due to enhanced adherence or simply reduced pharmacokinetic variability.

In a comprehensive review, Gonzales et al. proposed high tacrolimus IPV as a potential proxy for non-adherence [17] However, they also acknowledged that most supporting studies focused on the early post-transplant period, with limited evidence regarding the long-term predictive value of tacrolimus IPV.

While non-adherence is frequently cited as a major contributor to poor outcomes in kidney transplantation [34], several studies attributing poor outcomes to non-adherence did not directly measure adherence, limiting the strength of their conclusions. In addition, variability in CNI exposure also arises from multiple sources beyond patient behavior, including food-related changes in bioavailability, drug–drug interactions, variability in absorption and metabolism, epigenetic factors [35, 36], and clinical decision making. In routine practice, clinician-driven dose adjustments in response to intercurrent infections, rejection episodes, adverse effects, or evolving therapeutic targets are an integral component of therapeutic drug monitoring and are expected to generate variability in trough concentrations independently of adherence. This multidimensional nature of CNI variability likely explains, at least in part, why C_0_ and IPV performed poorly as proxies for adherence in our study. Rather than reflecting adherence alone, CNI exposure metrics integrate biological, pharmacokinetic, and clinical factors, thereby limiting their specificity as adherence markers when used in isolation.

Despite a consensual definition of adherence, the absence of a universally accepted gold standard for its measurement contributes to the heterogeneity of findings in the literature. As emphasized by several authors, identifying non-adherence reliably requires a multimethod approach, as each individual method -including pharmacokinetic monitoring- has well-documented limitations [13, 32, 37].

The association between high IPV of CNI C_0_ and poor clinical outcomes has been widely reported in kidney transplantation. Several studies have shown that patients with high IPV are at increased risk of rejection and graft loss, supporting the value of CNI therapeutic drug monitoring as a practical and accessible tool for identifying patients at risk [13, 38–40]. However, not all studies have confirmed this association. For instance, some authors found no significant correlation between high IPV and graft rejection [9] highlighting the complexity of interpreting IPV in isolation.

In our study, patients with lower IPV (CV <30%) tended to have better rejection-free survival at 3 years post-transplantation, although the difference did not reach statistical significance. This trend is consistent with the prevailing literature in kidney transplantation, where high IPV is generally associated with adverse outcomes. By contrast, evidence for this association is less consistent in liver transplantation, where pharmacokinetic variability appears to have a more limited prognostic value [33, 41].

One of the main strengths of our study is the longitudinal evaluation of both adherence and CNI C_0_ from the first month up to 1 year post-transplantation. While previous studies have often excluded the early post-transplant period due to greater pharmacokinetic fluctuations, particularly during the first 3 months [42]*,* our approach allowed for a more comprehensive characterization of adherence profiles and variability in drug exposure over time. By including this early period, we captured clinically relevant fluctuations in C_0_ that may influence long-term outcomes. Notably, CNI trough levels were collected as part of routine follow-up, regardless of the underlying clinical context, which may partly explain the observed variability and the difference in subtherapeutic C_0_ at 1 month post-transplant between adherent and non-adherent patients. In addition, the early post-transplant period is characterized by greater clinical instability and frequent dose adjustments, which could further contribute to these findings. Furthermore, information on medication prescriptions or dispensing history was not available, limiting our ability to distinguish pharmacokinetic variability related to clinical management from variability related to medication-taking behavior. Another strength lies in the granularity of our dataset: each patient contributed a large number of C_0_ measurements, which improves the accuracy of IPV estimates, in line with published recommendations [17]. Moreover, our analysis encompassed both tacrolimus and cyclosporine, offering a broader view of variability patterns across the two most commonly used CNIs in kidney transplantation. This dual-CNI perspective enhances the generalizability of our findings, whereas many prior studies focused on only one agent.

Our study also has some limitations. A key limitation is the use of a single method to assess adherence -namely, the MMAS-4 self-reported questionnaire. While widely used, self-reported tools are inherently subject to recall bias and social desirability effects. Although the literature emphasizes the value of combining multiple adherence assessment methods, such cross-validation is rarely implemented. To address this limitation, a subgroup of patients from the EPIGREN cohort also underwent face-to-face interviews conducted by a trained pharmacologist. In this subset, adherence estimates from MMAS-4 and interview-based assessments were highly concordant, with strong sensitivity and specificity, supporting the reliability of our primary adherence measure. Repeated administration of self-reported questionnaires may reduce response rates and data quality over time; however, the very short format of the MMAS (four items) likely limited, though did not fully eliminate, attrition-related bias.

Another limitation concerns the time frame of exposure assessment. While adherence trajectories were modeled over a three-year period, CNI exposure and variability were analyzed only during the first year. This may have limited our ability to capture late changes in pharmacokinetics or adherence behavior. As this was an observational study, causal relationships cannot be established, and residual confounding remains possible despite robust cohort design and analytic approaches.

Adherence was analyzed as a longitudinal and dynamic behavior, allowing changes over time to be captured regardless of their underlying causes, including intercurrent clinical events. However, the complex bidirectional relationship between post-transplant complications and adherence was not formally evaluated in this study.

Moreover, this study is based on real-world cohort data, and the exact timing of drug intake and blood sampling was not systematically recorded, which may have introduced variability in trough concentration measurements. Although samples were routinely collected immediately before the morning dose under standardized clinical practice, residual variability related to dosing habits cannot be fully excluded and could not be adjusted for in the analyses.

Finally, analyses conducted beyond 6 months should be interpreted descriptively rather than as supporting formal statistical comparisons, due to the reduced sample size and the progressive stabilization of CNI exposure and therapeutic drug monitoring practices over time [42, 43]. The absence of a formal sample size calculation, combined with progressive attrition over time, limits statistical power in later follow-up periods. Despite these limitations, the study provides valuable real-world insights into post-transplant outcomes in kidney transplant recipients.

In conclusion, no significant association was observed in this study between adherence -assessed both cross-sectionally and longitudinally using the MMAS-4- and CNI exposure parameters, including C_0_ and IPV, during the first year following kidney transplantation. Although therapeutic drug monitoring (TDM) may help identify isolated episodes of non-adherence -particularly in cases of markedly low C_0_- it remains insufficient for capturing adherence patterns over time. Our findings support the consensus that characterizing adherence requires a multimodal approach, integrating pharmacokinetic data with validated patient-reported measures and clinical interviews.

We also confirmed that high CNI variability is associated with a reduction in rejection-free survival, reinforcing the prognostic value of TDM for risk stratification beyond its role in dose individualization. These results underscore the importance of interpreting TDM in context, and not as a standalone indicator of adherence. Our findings also highlight the need for the development of integrated, scalable adherence monitoring strategies that can be feasibly implemented in routine care. Future work should explore combining digital adherence tools, behavioral interventions, and pharmacometric modeling to better identify at-risk patients and guide personalized adherence support.

## Data Availability

The original contributions presented in the study are included in the article/Supplementary Material, further inquiries can be directed to the corresponding author.
