# Supplementary material for: CNI Trough Variability Does Not Reliably Reflect Medication Adherence: Insights From a 3-Year Follow-Up Study
Source: Transpl Int. 2026 Mar 2;39:15718. doi: 10.3389/ti.2026.15718 (PMC12989446; doi:10.3389/ti.2026.15718)

Supplementary material

Morisky Medication-Taking Adherence Scale-MMAS (4-item)

English Version
1. Do you ever forget to take your medication?
2. Do you ever have problems remembering to take your medication?
3. When you feel better, do you sometimes stop taking your medication?
4. Sometimes if you feel worse when you take your medication, do you stop taking it?

MMAS-4 scoring: 1 point for each question respond as yes

MMAS-4 interpretation:
• 0 = High adherence
• 1-2= Medium adherence
• 3-4 = Low adherence

French Version
1 Vous arrive-t-il d'oublier de prendre votre traitement ?
2 Avez-vous parfois des difficultés à vous rappeler de prendre votre traitement ?
3 Quand vous vous sentez mieux, vous arrive-t-il d'arrêter de prendre votre traitement ?
4 Parfois, si vous vous sentez moins bien quand vous prenez votre traitement, arrêtez-vous de le prendre ?

Grille de cotation du MMAS-4 : 1 point par réponse « oui »

Interprétation du score MMAS-4 :
• 0 = Adhésion élevée
• 1-2 = Adhésion moyenne
• 3-4 = Faible adhésion

Adherence trajectories over time, identified using a mixed-effects modeling framework with latent processes and latent classes

Adherence trajectories after transplantation were analyzed using mixed models with latent processes and latent classes, implemented with the lcmm R package.

The modeling framework, previously described by our group, combines a measurement model and a structural model.

The measurement model links the observed adherence score to an underlying latent variable through an appropriate link function. Because MMAS-4 scores are non-Gaussian, a link function was used to transform the observed scores into a continuous Gaussian latent variable representing adherence.

The structural model describes the evolution of the latent adherence variable and identifies subgroups of patients (latent classes) sharing similar longitudinal adherence trajectories. Inter-patient heterogeneity was captured using a mixed model with latent classes. Covariates were first screened and subsequently included in the structural model if significantly associated with adherence trajectories.

Only patients with at least two adherence measurements were included in the analysis.

Adherence was measured at each study visit, generating longitudinal adherence profiles for each patient. All individual scores were included in the model, which allows handling of incomplete longitudinal data under a missing-at-random assumption. Model parameters were estimated using maximum likelihood.

Two latent classes were identified, corresponding to patients with predominantly adherent or non-adherent trajectories over time.

Classification was based on the overall trajectory of adherence scores rather than on individual MMAS-4 cutoffs at single time points.

This approach allows dynamic patient classification according to adherence evolution and provides a robust framework to explore factors associated with longitudinal adherence patterns after transplantation.

Supplementary Table S1. Distribution of calcineurin inhibitor trough concentrations (C0, µg/L) by transplant center

Supplementary Table S1a: Tacrolimus

CENTRE	Mean	Median	Q1	Q3	IQR
**AM**	9.6	9.3	7.3	11.5	4.2
**BX**	8.8	8.4	6.8	10.5	3.7
**LM**	9.5	9.1	7.5	10.9	3.4
**PO**	8.1	8.0	6.7	9.3	2.6
**RO**	8.0	7.7	6.0	9.8	3.8
**TL**	7.8	7.4	5.5	10.0	4.5
**TR**	9.3	9.2	7.5	10.7	3.2

Supplementary Table S1b: Cyclosporine

CENTRE	Mean	Median	Q1	Q3	IQR
**AM**	204	194	146	253	107
**BX**	155	146	113	189	76.0
**LM**	170	152	125	197	71.8
**PO**	158	149	126	187	61.0
**RO**	123	127	78.0	162	84.0
**TL**	140	126	82.5	169	86.5
**TR**	126	110	77.0	182	105

Supplementary Table S2: concomitant immunosuppressive therapies by center

Immunosuppressive therapies	Overall N = 619[1]	AM N = 98[1]	BX N = 227[1]	LM N = 108[1]	PO N = 10[1]	RO N = 97[1]	TL N = 69[1]	TR N = 10[1]	p
Patients on MMF	603 (97%)	98 (100%)	219 (96%)	108 (100%)	9 (90%)	93 (96%)	66 (96%)	10 (100%)	0.11[2]
CNI									<0.01[2]
ciclo	118 (19%)	49 (50%)	34 (15%)	18 (17%)	8 (80%)	2 (2.1%)	7 (10%)	0 (0%)	
switch	48 (7.8%)	8 (8.2%)	16 (7.0%)	15 (14%)	0 (0%)	7 (7.2%)	0 (0%)	2 (20%)	
tacro	453 (73%)	41 (42%)	177 (78%)	75 (69%)	2 (20%)	88 (91%)	62 (90%)	8 (80%)	

[1] n (%)
[2] Pearson's Chi-squared test

Hide

Supplementary Figure S1: Inter-center variability of calcineurin inhibitor trough concentrations during follow-up

Boxplots show the distribution of CNI C_0 by transplant center at each follow-up visit (M1, M3, M6, M9, and M12). Upper panel: tacrolimus; lower panel: cyclosporine.
Boxes represent the interquartile range, the horizontal line indicates the median, and whiskers extend to 1.5 times the interquartile range; individual observations are shown as open circles. Black dots indicate mean values. Inter-center comparisons were performed at each time point; corresponding p-values are reported in the text.

Tacrolimus

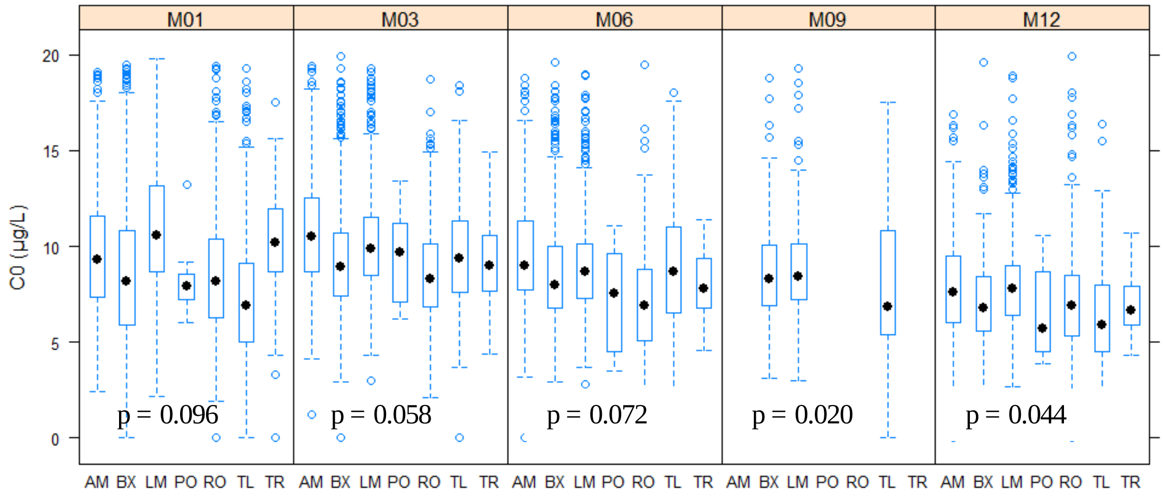

Cyclosporine

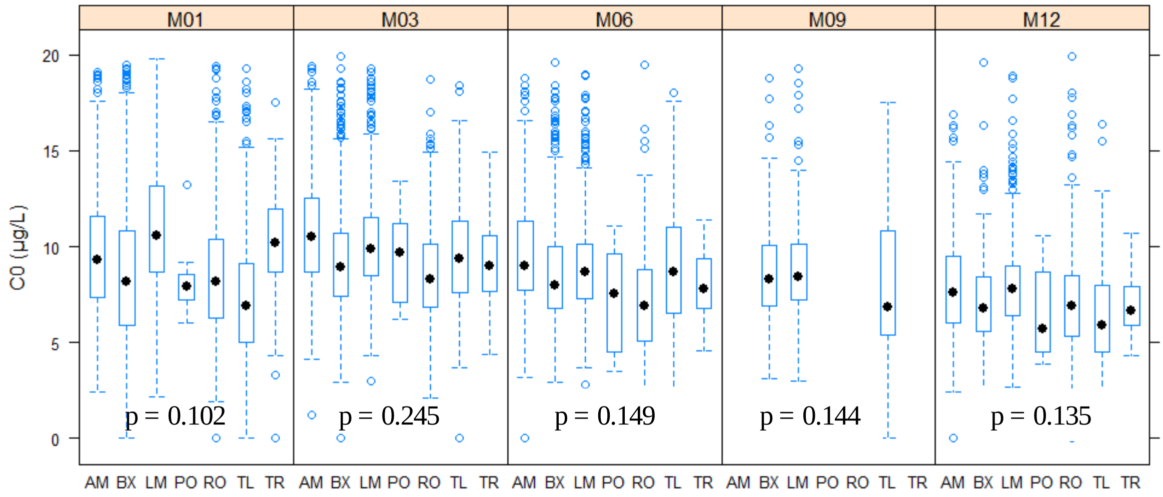

Supplement: Supplementary file 1 [file DataSheet1.pdf]
